# Prevalence, molecular typing, and antimicrobial resistance of bacterial pathogens isolated from ducks

**DOI:** 10.14202/vetworld.2019.677-683

**Published:** 2019-05-18

**Authors:** Hamza M. Eid, Abdelazeem M. Algammal, Wael K. Elfeil, Fatma M. Youssef, Sawsan M. Harb, Ehab M. Abd-Allah

**Affiliations:** 1 Department of Bacteriology, Mycology and Immunology, Faculty of Veterinary Medicine, Suez Canal University, Ismailia 41522, Egypt; 2 Department of Avian and Rabbit Medicine, Faculty of Veterinary Medicine Suez Canal University, Ismailia 41522, Egypt; 3 Department of Clinical Pathology, Animal Health Research Institute, Agriculture Research Center, Giza 12618, Egypt; 4 Veterinary Hospital, Faculty of Veterinary Medicine, Zagazig University, Zagazig 44511, Egypt

**Keywords:** Antibiotic sensitivity, duck, *Escherichia coli*, *Pasteurella multocida*, polymerase chain reaction, *Staphylococci*

## Abstract

**Aim:**

This study aimed to investigate the prevalence of different bacterial species affecting ducks as well as demonstrating the antimicrobial susceptibility and molecular typing of the isolated strains.

**Materials and Methods:**

A total of 500 samples were randomly collected from different duck farms at Ismailia Governorate, Egypt. The collected samples were subjected to the bacteriological examination. Polymerase chain reaction (PCR) was applied for amplification of *Kmt1* gene of *Pasteurella multocida* and X region of protein-A (*sp*A) gene of the isolated *Staphylococcus aureus* strains to ensure their virulence. The antibiotic sensitivity test was carried out.

**Results:**

The most common pathogens isolated from apparently healthy and diseased ducks were *P. multocida* (10.4% and 25.2%), *Escherichia coli* (3.6% and 22.8%), *Staphylococcus epidermidis* (10% and 8.8%), *Pseudomonas aeruginosa* (2% and 10%), and *Proteus vulgaris* (0.8% and 10%), respectively. In addition, *S. aureus* and *Salmonella* spp. were isolated only from the diseased ducks with prevalence (12.2%) and (2.8%), respectively. Serotyping of the isolated *E. coli* strains revealed that 25 *E. coli* strains were belonged to five different serovars O1, O18, O111, O78, and O26, whereas three strains were untypable. *Salmonella* serotyping showed that all the isolated strains were *Salmonella* Typhimurium. PCR revealed that four tested *P. multocida* strains were positive for *Kmt1* gene with specific amplicon size 460 bp, while three strains were negative. In addition, all the tested *S. aureus* strains were positive for *sp*A gene with specific amplicon size 226 bp. The antibiotic sensitivity test revealed that most of the isolated strains were sensitive to enrofloxacin, norfloxacin, and ciprofloxacin.

**Conclusion:**

*P. multocida* is the most predominant microorganism isolated from apparently healthy and diseased ducks followed by *E. coli* and *Staphylococci*. The combination of both phenotypic and genotypic characterization is more reliable an epidemiological tool for identification of bacterial pathogens affecting ducks.

## Introduction

Virulent bacteria are incriminated in huge economic casualties in the duck industry globally. Bacterial diseases cause higher mortality rates in ducks more than viral diseases. The mortality rates and bacterial diseases have been expanded worldwide [1-2]. Multiple of bacterial pathogens including *P*. *multocida*, *Escherichia coli, Staphylococci*, *Pseudomonas aeruginosa*, and *Salmonella* had become the major threats of duck health globally. Fowl cholera, occurred by *P. multocida*, remains one of the main problems of poultry worldwide [3]. It is a contagious disease of ducks, causing huge losses in the duck industry. The incidence of fowl cholera carriers in apparently healthy ducks could be 63%, while the mortality rate could reach 50% [4]. Moreover, *Staphylococcus aureus* is responsible for a broad spectrum of clinical signs in poultry including suppurative dermatitis, suppurative arthritis, and septicemic lesions [5-6]. *P. aeruginosa* is an opportunistic microorganism which causes several problems in ducks such as septicemia, diarrhea, respiratory manifestation, lameness, and conjunctivitis, and also, *E. coli* causes a wide variety of problems in ducks at different ages, but the most dangerous illness occurs at 2-6 weeks of age and mortality rates reach up to 43% [7,8]. One of the most important duck diseases is Salmonellosis. The disease is mainly showed an acute form at 3 weeks of age; the rate of chronic form in infected ducks is ranged from 0 to 66.7% in various flocks according to the age at *Salmonella* infection [4]. Various diseases affecting ducks may have common clinical manifestation and pathological lesions and its severity associated with the structure of duck immune system which differs from chickens and other vertebrates [8-10]. Moreover, ducks can be infected with two or more of these bacterial pathogens [9,11]. Polymerase chain reaction (PCR) is a highly sensitive technique used to detect different specific pathogens in the clinical samples. Many PCR assays have been developed for the detection and identification of duck bacterial pathogens [12].

This study aimed to investigate the prevalence of bacterial pathogens affecting ducks as well as molecular typing of the most pathogenic strains and determination of antibiotic sensitivity of the identified strains.

## Materials and Methods

### Ethical approval

Handling of ducks and laboratory animals was performed according to the Animal Ethics Review Committee of Suez Canal University, Egypt.

### Sampling

As illustrated in Table-1, 500 samples were randomly collected from 100 apparently healthy ducks (50 alive and 50 freshly slaughtered ducks) and 100 diseased ducks (50 alive and 50 freshly died and emergency slaughtered ducks) from commercial farms and traditional slaughterhouses at Ismailia Governorate, Egypt. Tracheal swabs and internal organs from freshly died and slaughtered ducks were collected. Samples were collected in peptone water (Oxoid, USA) under the complete aseptic conditions and rapidly transported to the lab for bacteriological examination.

**Table-1:** Type and number of collected samples from examined ducks.

Type of samples	Duck condition			

	Apparently healthy*(n=100)		Diseased ducks (n=100)	
	
	Live (n=50)	Freshly slaughtered (n=50)	**Live (n=50)	***Freshly dead and emergency slaughter (n=50)
Tracheal swab	50	-	50	-
Heart blood	-	50	-	50
Lung	-	50	-	50
Liver	-	50	-	50
Spleen	-	50	-	50
Total	50	200	50	200

* Apparently healthy birds were shown normal feed intake, smooth non-broken feathers, shiny eyes, and lack of any abnormal discharges from body orifice and no gross abnormalities.

** Diseased ducks were suffered from respiratory distress and diarrhea.

*** Postmortem examination revealed pneumonia, airsacculitis, and liver congestion with necrotic foci

### Bacteriological examination

#### Direct microscopical examination

Blood smears were prepared from heart blood then subjected to microscopical examination. Furthermore, the crushing of necrotic liver tissue between two slides was carried out, fixed by heating, stained by Giemsa stain, and examined microscopically for the detection of *P. multocida* [13].

#### Bacterial isolation and identification

The collected samples were inoculated in brain heart infusion broth and incubated aerobically at 37°C for 24 h. A loopful from incubated brain heart infusion broth was streaked onto nutrient agar, blood agar, mannitol salt agar, MacConkey’s agar, and eosin methylene blue agar plates then incubated for 24 h at 37°C. Separate pure colonies were picked up and inoculated on slope agar, then incubated at 37°C for 24 h, and then left for biochemical identification. Bacterial colonies were identified morphologically by using Gram’s stain as well as biochemically using methods described by Quinn *et al*. [13].

### Serotyping of *E. coli* strains

The isolated *E. coli* strains were subjected to serological identification (slide agglutination test) according to Edwards and Ewing [14]; using *E. coli* polyvalent and monovalent antisera.

### Serotyping of *Salmonella* isolates

Serodiagnosis of the isolated *Salmonella* strains was carried out using polyvalent (O) and monovalent antisera kit (Dade Behring Marburg GmbH–USA) D-35001, according to Grimont and Weill [15].

### Pathogenicity test for *P. multocida* strains

The pathogenicity test was carried out according to the methods described by Levy *et al*. [16]. Five rabbits (4 weeks age) were involved, 0.5 ml of whole culture (*P. multocida*) was injected (I/P) in rabbits. Rabbits were observed for 2 days post-inoculation. *P*. *multocida* was reisolated from internal organs of the examined rabbits.

### Molecular typing of *Kmt1* gene of *P. multocida* and X region of protein-A (spA) gene of *S. aureus* strains

#### Extraction of DNA from isolates using the boiling method [17]

About 1 ml of bacterial broth culture was centrifuged at 5000 rpm for 5 min, and then the supernatant was removed. Pellets were resuspended in 1 ml distilled water, followed by centrifugation at 5000 rpm/5 min, and then resuspended in 200 µl distilled water. The suspension was boiled for 10 min, then placed in ice for 5 min, and then centrifuged at 10,000 rpm for 5 min. The supernatant (contain the bacterial DNA) was transferred to a fresh tube. The concentration and purity of the extracted DNA were determined by estimating the optical density at wavelengths of 260 and 280 nm using the spectrophotometer. The concentration was calculated as follows: OD260 = 50 ug/ml, purity of DNA = OD260 nm/OD280 nm.

#### Polymerase chain reaction

Primers used in PCR (Metabion, Germany) (Table-2)

**Table-2:** Oligonucleotide primers sequences used in PCR.

Target gene	Primers sequences	Amplicon (bp)	Reference
*Kmt1*			
For	ATCCGCTATTTACCCAGTGG	460	[19]
Rev	GCTGTAAACGAACTCGCCAC		
*sp*A			
For	TCAACAAAGAACAACAAAATGC	226	[20]
Rev	GCTTTCGGTGCTTGAGATTC		

PCR=Polymerase chain reaction, *sp*A=X region of protein-A

DNA samples were tested in 50 μl reaction volume in a 0.2 ml PCR tube, containing PCR buffer, dNTPs(dATP, dGTP, dCTP and dTTP) 200 μM for each; two primer pairs each at 50 picomol/reaction and 1.25 unite of Taq DNA polymerase. A control negative reaction with no template DNA was also used. Thermal cycling was carried out in a programmable thermal cycler (Coy Corporation, Grass Lake, USA) [18].

PCR cycling condition

PCR protocol of *Kmt1* gene was done according to the OIE 2012 [19] manual and *sp*A gene according to Wada *et al*. [20]; Denaturation at 94°C for 1 min (Annealing at 55°C fo *r Kmt*1 gene and at 60°C for *sp*A gene for 1 min); Extension at 72°C for 1 min run for 30 cycles with 10 min final extension at 72°C.

Screening of PCR products

About 10 µl of the amplified PCR product was analyzed by electrophoresis on a 2% agarose gel stained with 0.5 µg of ethidium bromide/ml. Electrophoresis was carried out in 1× TAE buffer at 80 volts for 1 h. Gels were visualized under ultraviolet transilluminator (UVP, UK) and photographed [21].

### Antibiotic susceptibility testing

The susceptibility to 12 different antimicrobial agents was tested according to the instructions of NCCLS [22] manuals; using disk diffusion technique depending on the diameter of the inhibition zone [23]. The following antibiotics were tested; enrofloxacin (5 μg), norfloxacin (10 μg), ciprofloxacin (5 μg), gentamycin (10 μg), amoxicillin (25 μg), neomycin (30 μg), erythromycin (15 μg), streptomycin (10 μg), oxytetracycline (30 μg), trimethoprim-sulfamethoxazole (25 μg), ampicillin (10 μg), and penicillin (10 I.U); (Oxoid, USA).

## Results

### Postmortem examination

Postmortem examination of diseased birds revealed a picture of septicemia, blood vascular congestion, hemorrhagic enteritis, swollen, and sometimes congested liver with multiple necrotic foci on the parietal surface. Trachea and lungs were severely congested and hemorrhagic, and serofibrinous exudates were observed in the lung, liver, and heart.

### Bacteriological examination

As shown in Tables-3 and 4, the bacteriological examination revealed that the most predominant strains isolated from apparently healthy and diseased ducks were *P. multocida* (10.4% and 25.2%), *E. coli* (3.6% and 22.8%), *Staphylococcus epidermidis* (10% and 8.8%), *P. aeruginosa* (2% and 10%), and *Proteus vulgaris* (0.8% and 10%), respectively. In addition, *S. aureus* and *Salmonella* spp. were isolated only from the diseased ducks with prevalence (12.2%) and (2.8%), respectively.

**Table-3:** Prevalence of the isolated bacterial strains from apparently healthy ducks in relation to the total number of samples.

Bacterial species	Total number of ducks	Tracheal swabs (n=50)	Total number of slaughter ducks (n=50)				Total number of isolates/total number of samples (n=250)

			Heart (50)	Lung (50)	Liver (50)	Spleen (50)	
					
		n (%)	n (%)	n (%)	n (%)	n (%)	n (%)
*P. multocida*	15	10 (20)	5 (10)	5 (10)	3 (6)	3 (6)	26 (10.4)
*E. coli*	3	0 (0)	3 (6)	3 (6)	3 (6)	0 (0)	9 (3.6)
*S. epidermidis*	10	5 (10)	5 (10)	5 (10)	5 (10)	5 (10)	25 (10)
*P. aeruginosa*	2	1 (2)	1 (2)	1 (2)	1 (2)	1 (2)	5 (2)
*P. vulgaris*	2	0 (0)	0 (0)	0 (0)	2 (4)	0 (0)	2 (0.8)
Total	32	16 (32)	14 (28)	14 (28)	14 (28)	9 (18)	67 (26.8)

*P*. *multocida*=*Pasteurella* *multocida*, *E*. *coli*=*Escherichia* *coli*, *S*. *epidermidis*=*Staphylococcus* *epidermidis*, *P*. *aeruginosa*=*Pseudomonas* *aeruginosa*, *P*. *vulgaris*=*Proteus* *vulgaris*

**Table-4:** Prevalence of the isolated bacterial strains from diseased ducks in relation to total number of samples.

Bacterial species	Total number of ducks	Tracheal swabs (n=50)	Total number of slaughter ducks (n=50)				Total number of isolates/total number of samples (n=250)

			Heart (50)	Lung (50)	Liver (50)	Spleen (50)	
					
		n (%)	n (%)	n (%)	n (%)	n (%)	n (%)
*P. multocida*	30	19 (38)	11 (22)	11 (22)	11 (22)	11 (22)	63 (25.2)
*E. coli*	25	14 (28)	11 (22)	11 (22)	11 (22)	10 (20)	57 (22.8)
*S. aureus*	11	5 (10)	6 (12)	6 (12)	6 (12)	5 (10)	28 (12.2)
*S. epidermidis*	10	5 (10)	5 (10)	5 (10)	4 (8)	3 (6)	22 (8.8)
*P. aeruginosa*	10	5 (10)	5 (10)	5 (10)	5 (10)	5 (10)	25 (10)
*S. Typhimurium*	5	0 (0)	0 (0)	0 (0)	5 (10)	2 (4)	7 (2.8)
*P. vulgaris*	9	2 (4)	5 (10)	5 (10)	7 (14)	6 (12)	25 (10)
Total	100	50 (100)	43 (86)	43 (86)	49 (98)	42 (84)	227 (90.8)

*P*. *multocida*=*Pasteurella* *multocida*, *E*. *coli*=*Escherichia* *coli*, *S*. *epidermidis*=*Staphylococcus* *epidermidis*, *P*. *aeruginosa*=*Pseudomonas* *aeruginosa*, *P*. *vulgaris*=*Proteus* *vulgaris*, *S*. *aureus*=*Staphylococcus* *aureus*, *S*. *Typhimurium*=*Salmonella* *Typhimurium*

### Serotyping of *E. coli* and *Salmonella* strains

As shown in Table-5, serological typing of 28 *E. coli* strains revealed that 25 strains were belonged to five different serovars O1, O18, O111, O78, and O26; moreover, three strains were untypable (isolated from diseased ducks). In addition, *Salmonella* serotyping proved that all the isolated *Salmonella* strains from the examined ducks were *Salmonella* Typhimurium.

**Table-5:** Serotyping of the isolated *E. coli* strains from apparently healthy and diseased ducks.

Serotype of *E. coli*	Apparently healthy ducks freshly slaughtered (*n*=50)	Diseased ducks		Total

		Live (n=50)	Slaughtered (n=50)	
			
	n (%)	n (%)	n (%)	n (%)
O1	1 (2)	3 (6)	2 (4)	6 (21.42)
O18	2 (4)	2 (4)	1 (2)	5 (17.85)
O111	-	2 (4)	1 (2)	3 (10.71)
O78	-	5 (10)	2 (4)	7 (25)
O26	-	1 (2)	3 (6)	4 (14.28)
Untypable	-	1 (2)	2 (4)	3 (10.71)
Total	3 (6)	14 (28)	11 (22)	28 (100)

*E. coli*=*Escherichia coli*

### The pathogenicity of *P. multocida*

The pathogenicity of the isolated *P. multocida* strains was tested experimentally in five rabbits at 4 weeks age by inoculation of 0.5ml (I/P) of *P. multocida* broth culture, the death of inoculated rabbits usually occurs within 18-24 h. The examined died rabbits showed septicemic carcass, congested internal organs, and hemorrhage from the nose.

### Molecular typing of *P. multocida* and *S. aureus*

**Figure-1:**
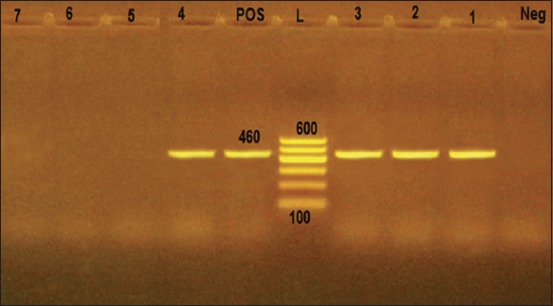
Electrophoretic pattern of *Kmt1* gene polymerase chain reaction assay. Lane L: 100 bp DNA Ladder, Lane Pos: Control positive strain (reference strain kindly given by the Animal Health Research Institute, Dokki, Egypt). Lane Neg: Control negative. Lanes 1-4: Positive isolated *Pasteurella multocida* strains for *Kmt1* gene at 460 bp. Lanes 5-7: Negative isolated *P. multocida* strains for *Kmt1* gene.

**Figure-2:**
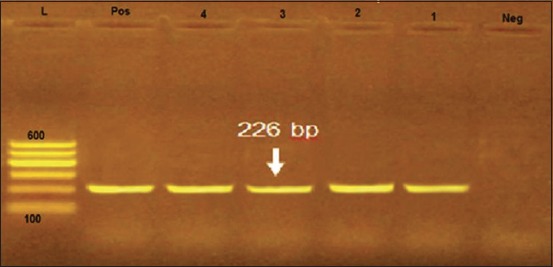
Electrophoretic pattern of protein A gene polymerase chain reaction assay. Lane L: 100 bp DNA ladder; Lane Pos: Control positive strain (reference strain kindly given by the Animal Health Research Institute, Dokki, Egypt). Lane Neg: Control negative. Lanes 1-4: Positive *Staphylococcus aureus* strains for X region of protein-A gene at 226 bp.

In the present study, PCR protocol was used for amplification and detection of *Kmt1*gene in the isolated *P. multocida* strains. As shown in Figure-1, four examined isolated strains were positive for *Kmt1* gene with specific amplicon size 460 bp, while three other isolated strains were negative. In addition, PCR protocol was used for amplification and detection of *sp*A gene in the isolated *S. aureus* strains. Figure-2 illustrated the positive amplification of 226 bp fragment of *sp*A gene from the extracted DNA of the isolated *S. aureus* strains, where all the tested strains were positive for *sp*A gene.

### Antibiotic susceptibility testing

As shown in Table-6, the antimicrobial susceptibility testing revealed that the isolated *P. multocida, S. aureus*, and *P. aeruginosa* strains were found to be highly sensitive to enrofloxacin, norfloxacin, and ciprofloxacin. The isolated *S. aureus* strains were highly resistant to ampicillin, amoxicillin, and penicillin, while the isolated *P. aeruginosa* strains were highly resistant to penicillin, streptomycin, erythromycin, and sulfamethoxazole-trimethoprim. In addition, *E. coli* serotypes and *S*. Typhimurium strains were highly sensitive to norfloxacin, ciprofloxacin, and enrofloxacin. *S*. Typhimurium strains were resistant to amoxicillin and erythromycin, while *E. coli* serotypes were resistant to penicillin, streptomycin, and ampicillin.

**Table-6:** Antimicrobial susceptibility of the bacterial isolates from ducks (shown in percentage).

Antimicrobial agent	*P. multocida*			*S. aureus*			*P. aeruginosa*			S. Typhimurium			*E. coli* serotypes				
				
	S	I	R	S	I	R	S	I	R	S	I	R	O1	O18	O78	O26	O111
Enrofloxacin	100	-	-	100	-	-	100	-	-	100	-	-	S	S	S	S	S
Norfloxacin	100	-	-	100	-	-	100	-	-	100	-	-	S	S	S	S	S
Ciprofloxacin	100	-	-	100	-	-	100	-	-	100	-	-	S	S	S	S	S
Erythromycin	60	40	-	-	20	80	-	20	80	-	28.5	71.5	S	S	S	I	I
Streptomycin	20	80	-	-	10	90	-	10	90	28.5	71.5	-	R	R	R	R	R
Ampicillin	20	80	-	-	-	100	-	30	70	-	71.5	28.5	R	R	R	R	R
Amoxicillin	70	30	-	-	-	100	80	20	-	-	28.5	71.5	I	R	R	R	R
Penicillin	90	10	-	-	-	100	-	-	100	57	43	-	R	R	R	R	R
Gentamycin	90	10	-	60	20	20	60	20	20	86	14	-	S	S	S	I	I
Neomycin	40	60	-	80	20	-	80	20	-	86	14	-	R	S	I	I	R
Oxytetracycline	70	30	-	-	40	60	-	40	60	71.5	28.5	-	S	R	R	R	R
Trimethoprim-sulfamethoxazole	70	30	-	-	20	80	-	20	80	86	14	-	S	S	I	I	R

S=Sensitive, I=Intermediate, R=Resistant, *P*. *multocida*=*Pasteurella* *multocida*, *E*. *coli*=*Escherichia* *coli*, *P*. *aeruginosa*=*Pseudomonas* *aeruginosa*, *S*. *aureus*=*Staphylococcus* *aureus*, *S*. Typhimurium=*Salmonella* Typhimurium

## Discussion

Regarding the results shown in Tables-3 and 4, the bacteriological examination of 500 collected samples revealed the isolation of 67 bacterial strains (26.8%) from apparently healthy ducks as well as, 227 strains (90.80%) from the diseased ducks. These results are in agreement with those obtained by Rehab [24]. However, ducks are relatively resistant to certain diseases; there are many risk factors increase their susceptibility to infection such as bad management, poor sanitary conditions, malnutrition, overcrowding, and environmental stresses [25].

In the present study, the prevalence of *P. multocida* and *E. coli* was (10.4%) and (3.6%) in apparently healthy ducks, while in diseased ducks were (25.2%) and (22.8%), respectively.

Serological typing of the isolated *E. coli* strains revealed that 25 strains were belonged to five different serogroups including O1, O18, O111, O78, and O26; while three strains were serologically untypable (were isolated from diseased ducks) as shown in Table-5. These results are agreed with those obtained by Radad [26] and Abdel-Rahman *et al.*, [27]. *P. multocida* mainly inhabits the upper respiratory tract as a commensal or an opportunistic microorganism, but its virulence increases due to stress conditions, so the microorganism invades the lung tissues [28,29]. *E. coli* commonly inhabits the intestinal tract, but it often infects the respiratory tracts of birds in combination with infection by other microorganisms. These infections mainly affect the air sacs and the infections are referred to as chronic respiratory disease [1]. *P. multocida* infection was almost constantly followed by *E. coli* infection in poultry [30]. In the present study, the prevalence of *S. aureus*, *P. aeruginosa*, and *Salmonella* in diseased ducks was (12.2%), (10%), and (2.8%), respectively. Serotyping of *Salmonella* strains revealed that all the isolated strains were *S*. Typhimurium. These findings are agreed with those obtained by Mona *et al*. [25], Abdel-Rahman *et al*. [27], and Tawwab *et al*. [31]. *S. aureus* is mainly incriminated in the infection of the upper respiratory tract, especially when stress conditions increased [32]. Powerful toxins produced by *P. aeruginosa* are mainly incriminated in respiratory manifestation in poultry [33]. Salmonellosis is a common contagious disease of man and animal [34]. Mortality rates vary according to the degree of virulence and host immunity [5]. Results of the pathogenicity test in susceptible rabbits revealed that the isolated *P. multocida* strains were highly virulent and cause rabbit death within 24 h after I/P inoculation, which is accompanied by generalized septicemia. These results are agreed with those obtained by Fatma [35]. Pasteurellosis is a bacterial septicemic disease of rabbit, which affects different tissues and organs inducing pathological changes accompanied by septicemia [36]. In the present study, PCR protocol was used for amplification and detection of *Kmt1*gene in the isolated *P. multocida* strains. As illustrated in Figure-1, four examined strains were positive for *Kmt1* gene with specific amplicon size 460 bp, while three strains were negative. These results agreed with those obtained by Deressa *et al*. [37]. Furthermore, PCR protocol used for amplification and detection of *sp*A gene in the isolated *S. aureus* strains. Figure-2 illustrated the positive amplification of 226 bp fragment of *sp*A gene from the extracted DNA of the isolated *S. aureus* strains, where all the tested strains were positive for *sp*A gene; these results agreed with those obtained by Akineden *et al*. [38]. PCR used for amplifying specific target DNA sequences is an even more sensitive procedure either for confirming the diagnosis of the isolated microorganism or detection of specific genes that are responsible for the production of the virulence factors [39]. Regarding the results shown in Table-6, the antimicrobial susceptibility testing revealed that the isolated *P. multocida* strains were found to be highly sensitive to enrofloxacin, norfloxacin, and ciprofloxacin followed by penicillin and gentamycin. These results are agreed with those obtained by Balakrishnan and Roy [40] and disagree with those obtained by Akineden *et al*. [38]. *S. aureus* strains were found to be highly sensitive to enrofloxacin, norfloxacin, and ciprofloxacin and highly resistant to ampicillin, amoxicillin, and penicillin. Inactivation of penicillin resulted from the production of penicillinase enzyme by *S. aureus*, which causes the destruction of the beta-lactam ring of penicillin. The *blaZ* gene which is carried on S. aureus plasmid is mainly responsible for penicillin resistance[41]. In this study, *P. aeruginosa* strains were found to be highly sensitive to enrofloxacin, norfloxacin, and ciprofloxacin and were highly resistant to penicillin, streptomycin, erythromycin, and sulfamethoxazole-trimethoprim. In addition, *S. Typhimurium* strains were found to be highly sensitive to enrofloxacin, norfloxacin, and ciprofloxacin. These results are agreed with those obtained by Mona *et al*. [25], Abdel-Rahman *et al*. [27], and Tawwab *et al*. [31]; in this concern, Hanafy *et al*. [42] found that enrofloxacin was the most effective antibiotic against all strains (100%) of *P. aeruginosa*. The multiresistant property of *P. aeruginosa* may be attributed to the physicochemical properties of the cell rather than antibiotic inhibitory enzymes [43]. As regard to antimicrobial susceptibility of *E. coli* serotypes as shown in Table-6, all the isolated *E. coli* serotypes were highly sensitive to enrofloxacin, ciprofloxacin, and norfloxacin and were highly resistant to ampicillin. Fatma [35] recorded that the isolated *E. coli* serotypes were highly sensitive to enrofloxacin and highly resistant to ampicillin and streptomycin [41]. Enrofloxacin is frequently, used in the treatment of *E. coli* infection in poultry [44, 45].

## Conclusion

*P. multocida* is the most predominant microorganism isolated from apparently healthy and diseased ducks followed by *E. coli* and *Staphylococci*. Enrofloxacin, norfloxacin, and ciprofloxacin are the most effective antibiotics against different bacterial pathogens affecting ducks. Combination of genotypic and phenotypic characterization is more valuable as an epidemiological tool for identification of bacterial pathogens affecting ducks; moreover, PCR is a rapid and reliable tool used for confirming the virulence of the isolated strains.

## Authors’ Contributions

HME, AMA, FMY, WKE, SMH, and EMA involved in the conceptualization and design of the study. AMA, WKE, SMH, and EMA conducted the experiment and analyzed and interpreted the data. AMA, HME, FMY and WKE wrote and revised the manuscript. All authors reviewed, edited, and approved the final manuscript.
